# Revisited guidelines for metabolic tolerance tests in mice

**DOI:** 10.1038/s41684-024-01473-5

**Published:** 2024-11-25

**Authors:** Cedric Moro, Christophe Magnan

**Affiliations:** 1 Institute of Metabolic and Cardiovascular Diseases, INSERM, Team MetaDiab, Paul Sabatier University, UMR1297, Toulouse, France; 2 Unité de Biologie Fonctionnelle et Adaptative, Université Paris Cité, CNRS, Paris, France

**Keywords:** Type 2 diabetes, Experimental models of disease

## Abstract

Preclinical mouse models are extensively used in biomedical research to gain insight into disease mechanisms and to test new drug treatments. Glucose and insulin tolerance tests are simple experimental tests frequently used worldwide to assess glucose metabolism in mice. Various guidelines and methodological considerations have been published to help researchers standardize procedures and optimize research outcomes. Yet, there is still important experimental heterogeneity in the way these simple procedures are performed, with no real consensus on what the best practices are to achieve high-quality research and reproducible results. Here we critically examine several published guidelines and recent technical reports on how to perform these metabolic tests in laboratory mice and discuss the influence of various confounding factors on test results. We hope this work will help scientists establish more consensual guidelines for maximizing the relevance and clinical translation of studies using mouse models in metabolic research.

## Abstract (web summary)

In this Review, the authors examine current guidelines for metabolic tolerance tests in mice and provide a set of revisited recommendations to improve the reproducibility and clinical translation of the findings.

## Main

Glucose tolerance tests (GTT) and insulin tolerance tests (ITT) are by far the most common and simple metabolic tests used by researchers to assess glucose homeostasis and metabolic health in laboratory mice^1^. They are rapid and powerful screening methods to detect impaired carbohydrate metabolism, glucose intolerance and insulin resistance. Despite the substantial number of guidelines and technical reports published over the past 15 years to advise on how to conduct these metabolic tests, there are large differences in laboratory practices that can radically influence the outcomes of the tests, data interpretation and the reproducibility of the results. We therefore critically examine several guidelines and technical reports of the past 15 years to discuss the various methodological aspects of these metabolic tolerance tests (MTT) and the influence of confounding factors. We also provide a set of revisited guidelines describing what we believe are the best practices to perform these tests in laboratory mice.

## Experimental procedures

Comprehensive standard operating procedures and practical implementation protocols on how to perform MTT in laboratory rodents have been published elsewhere^2,3^. MTT include the widely used GTT and ITT. Other common MTT include gluconeogenesis tests (NGT) based on the intraperitoneal (i.p.) administration of pyruvate, glycerol, glutamine or alanine at various doses. In some cases, insulin secretion tests (IST) based on the administration of high bolus doses of glucose or arginine can be performed to assess pancreatic function. We have summarized the current recommendations for conducting these MTT (Table 1). From this table, it is striking to note that there is no consensus on fasting time duration, route and dose of administration of metabolic substrates, normalization method of the bolus dose, timing of plasma and/or tissue collection and calculation mode for data interpretation. However, all these factors can impede data interpretation and reproducibility within and between laboratories. Hereafter, we critically discuss basic considerations and common confounding factors for MTT in mice.

**Table 1:** Different MTT used in laboratory mice and their current guidelines

MTT	Fasting time	Dose; route; readout	References
Glucose tolerance (GTT)	5–6 h or overnight 14–18 h (for muscle phenotype)	1–2 g/kg LBM glucose; i.p., i.v. or oral; AUC glucose	Ayala et al.^27^
	5–6 h	1–2 g/kg BW glucose; i.p. or oral; AUC glucose (normalize dose to LBM when possible)	Alquier et al.^65^
	Overnight 14 h	1–3 g/kg BW glucose; oral; AUC glucose	Nagy et al.^66^
	6 h	2 g/kg BW glucose; i.p. or oral; iAUC glucose 3 g/kg LBM glucose; i.p. or oral; iAUC glucose	Virtue et al.^1^
Insulin sensitivity (ITT)	5–6 h	0.5–1.0 U/kg BW or LBM insulin; i.p.; AUC glucose	Ayala et al.^27^
	5–6 h	0.5–1.0 U/kg BW insulin; i.p.; AUC glucose (normalize dose to LBM when possible)	Alquier et al.^65^
	2–5 h	0.75 U/kg BW insulin; i.p.; inverse AUC	Nagy et al.^66^
	6 h	0.5–1.0 U/kg BW insulin; i.p.; AOC glucose	Virtue et al.^1^
Insulin secretion (IST)	5–6 h	3 g/kg BW glucose; i.p.; 0, 5 and 10 min blood glucose 0.3 g/kg BW l-arginine; i.p.; 0, 5 and 10 min blood glucose	Malakauskas et al.^67^
	5–6 h	1.0–2.5 g/kg BW l-arginine; i.p., i.v.; 0, 5 and 10 min blood glucose	Andrikopoulos et al.^8^
Gluconeogenesis (NGT)	6–12 h or overnight	2 g/kg BW pyruvate; i.p.; AUC glucose 2 g/kg BW glycerol; i.p.; AUC glucose 2 g/kg BW l-glutamine; i.p.; AUC glucose 0.5–2.0 g/kg BW alanine; i.p.; AUC glucose	Benede-Ubieto et al.^3^
Insulin sensitivity (HIEC)	5–6 h	HIEC in conscious mice; GIR and Rd	Ayala et al.^26^
Tissue-specific glucose uptake	6 h	Radioactive 2-deoxyglucose (8–10 µCi per mouse); combined to bolus injection of glucose (2 g/kg BW) i.p.; tissue collection at 30–160 min, Rg	Mauvais-Jarvis et al.^68^
	2 h	Radioactive 2-deoxyglucose (0.4 µCi/g mouse); combined to bolus injection of insulin (3 U/kg BW) i.p.; tissue collection at 30 min, Rg	Laurens et al.^37^
Tissue-specific insulin signaling	6 h	Insulin (10 U/kg BW); i.p.; tissue collection at 10 min, relative protein phosphorylation	Kienesberger et al.^61^

AOC, area of the curve; AUC, area under the curve; BW, body weight; GIR, glucose infusion rate; HIEC, hyperinsulinemic–euglycemic clamp; i.v., intravenous; Rd, rate of disappearance; Rg, rate of metabolic clearance of glucose.

## Basic considerations

### Genetic background

Previous work has demonstrated strain-dependent differences in metabolic traits when comparing various inbred mouse strains^4–6^. Different mouse strains can have varying baseline insulin sensitivities and metabolic rates, which can complicate the interpretation of results and comparisons across studies. Studies have reported that C57BL/6J mice develop more pronounced insulin resistance and obesity when on a high-fat diet (HFD) compared with 129/Sv mice, which show relatively preserved insulin sensitivity^5^. It was also observed that the C57BL/6J mouse strain exhibits greater blood glucose excursions compared with other strains during i.p. GTT, possibly due to lower glucose-stimulated insulin secretion^4^. Studies have also reported that this strain is highly susceptible to diet-induced glucose intolerance and shows a worsening of the metabolic status over time^6^.

In 2023, Karimkhanloo et al. provided an in-depth comparison of nonalcoholic steatohepatitis (NASH) pathology and deep-metabolic profiling in eight common inbred mouse strains (A/J, BALB/c, C3H/HeJ, C57BL/6J, CBA/CaH, DBA/2J, FVB/N and NOD/ShiLtJ)^7^. They report high interstrain variability in response to diet-induced metabolic dysfunction and NASH, with C57BL/6J, CBA and FVB/N being the most susceptible strains to NASH. Interestingly, they also show that C57BL/6J and FVB/N mice are more prone to diet-induced obesity and glucose intolerance than other strains. Diet-induced insulin resistance assessed by ITT seemed more pronounced in FVB/N mice. Thus, the investigators conclude that FVB/N is the mouse strain that most faithfully recapitulates human NASH pathophysiology, including the most predominant NASH-associated metabolic comorbidities^7^. In their study, the team used oral GTT (oGTT) 2 g/kg and i.p. ITT 0.75 U/kg after a 4 h fast. This work is in line with other studies that have previously reported a large interstrain variability in blood glucose and insulin responses during GTT^2,8–10^. In another study, Berglund et al. performed euglycemic, hypoglycemic and hyperglycemic clamps in four different mouse strains to study insulin secretion and the counter-regulatory response to hypoglycemia^5^. They observed that glucose-stimulated insulin secretion was blunted in FVB/N mice. FVB/N mice also showed relative hepatic insulin resistance and the highest counter-regulatory response to hypoglycemia. Among all strains, DBA/2 mice showed the weakest insulin action. These interstrain differences in metabolic responses highlight the importance of considering strain-specific characteristics when designing experiments and interpreting data. Collectively, these studies demonstrate that the choice of the mouse strain is an important factor to consider for optimizing study outcomes.

### Age

Most studies in the field of metabolism use rather young adult mice, typically between 8 and 16 weeks old, except when explicitly studying the effects of aging. It is well known that aging is a major risk factor of type 2 diabetes. Pioneer studies have shown that aging promotes glucose intolerance in mice, despite an increase in glucose-induced insulin secretion with age^11^. Aging also promotes insulin resistance in mice, as assessed through ITT^12^. Sometimes discrepant results have been reported for glucose tolerance^12^ or insulin resistance^13^ in old mice (>50 weeks). However, one study has reported impaired insulin sensitivity and reduced insulin clearance through hyperinsulinemic–euglycemic clamps in aged mice on chow diets and HFDs, independently of adiposity^13^. Therefore, age is a substantial confounding variable in metabolic research; experiments should be replicated on multiple cohorts of mice within a narrow age range to increase the reproducibility of the results.

### Sex

Sex differences in the prevalence of diabetes have been reported in humans and mice, with males being more susceptible than females^14,15^. However, researchers have predominantly used male mice in metabolic research because female mice usually display lower blood glucose levels and glucose excursions than males during GTT. Additional reasons for only including male mice in metabolic studies include housing cost and the difficulty to synchronize the estrus cycle in female mice. This practice introduces a bias because estrogens are well known to have beneficial effects on energy metabolism and insulin sensitivity^16^. However, experimental evidence suggests that the metabolic traits of female rats are no more variable than those of male rats^17,18^. In 2022, Kennard et al. refuted the common misconceptions that led to the exclusion of female mice from experimental protocols^19^. They demonstrated that, compared with males, female mice showed less variable GTT responses, which were not affected by the estrous cycle. Consequently, female mice are not less useful than male mice for investigating metabolic traits. These observations are in line with the decision of the National Institutes of Health to issue a mandate for considering sex as a biological variable in preclinical research, and a guide for the design of preclinical studies on sex differences in metabolism is available with specific recommendations^18^. It is therefore critical to include female mice in metabolic research to increase the translatability of the findings.

## Confounding factors

### Fasting duration

Since 2010, various guidelines articles have recommended a fasting duration of 6 h before MTT to minimize weight loss, arguing that this fast duration is sufficient to reduce blood glucose concentration, minimize gastric content and ensure gastric clearance of food consumed in the dark phase (Table 1). Yet, it is still common to find data from ITT or GTT performed after an overnight fast (14–18 h) in recently published studies, even in highly visible, generalist journals^20,21^. According to Kennard et al., overnight fast is still widely used by researchers, with a review of the literature using the keywords ‘glucose tolerance test and mouse’ indicating that 60% of 353 studies published between 2018 and 2019 still fasted mice overnight^19^. Overnight fasting before GTT is still recommended in recent methods papers and book chapters^22–24^. However, it has been repeatedly reported that overnight fasting promotes major weight loss, lean mass loss and liver glycogen depletion, thus preventing a proper glucose counter-regulatory response typically observed when blood glucose falls below 80 mg/dl (ref. ^25^). In a study comparing the effects of different factors on insulin sensitivity in C57BL/6J mice, Ayala et al. showed that overnight fasting (18 h) resulted in a substantial loss of total body mass, lean body mass (LBM) and fat mass (FM), as well as almost total depletion of hepatic glycogen compared with a 5 h fast^26^. In 2010, the National Institutes of Health Mouse Metabolic Phenotyping Center Consortium proposed guidelines to conduct metabolic tests of glucose homeostasis in mice^27^. Since then, 6 h fasts have been widely and routinely used by most investigators in the field of metabolism to determine whether fasting glucose levels fall within a normal range. Other laboratories have used empirically shorter fasting times (2–6 h) before MTT^28–30^. In 2021, we reassessed the optimal fasting time needed before ITT using a time course of fasting in C57BL/6J mice^31^. We reached the conclusion that 2 h fasting during the light phase is sufficient to reduce blood glucose variability and reduce insulin levels by ~40%, similarly to longer fast (≥4 h). By contrast, longer fasts were associated with time-dependent body-weight loss and a profound catabolic state in insulin-sensitive tissues such as liver and skeletal muscle^31^. This starvation state was associated with considerable liver glycogen depletion, which impaired the glucose counter-regulatory response to insulin injection during ITT. We further showed that 12 h overnight and 24 h fasting periods were associated with >10% body weight loss and muscle atrophy, thus promoting a marked state of insulin resistance. Kennard et al. also reached similar conclusions, showing that 16 h fasting impaired glucose tolerance in both male and female C57BL/6J mice compared with 6 h fasting^19^. Glucose tolerance was assessed by i.p. GTT 2 g/kg in their study. We have recently discussed the issue of optimal fasting time before ITT and GTT^32^. Although the influence of fasting duration on other MTT has not been tested yet, we believe that the physiological adaptations induced by fasting are unlikely to differ meaningfully from one MTT to another. For instance, weight loss, robust induction of hepatic lipid catabolism pathways, and induction of a muscle atrophy program and muscle wasting are already detectable after a 4 h fast^31^.

In an 1986 study assessing gastric emptying in Charles River rats, Ramirez demonstrated a very fast gastric emptying of glucose administered either by intragastric feeding or by oral gavage^33^. Gastric content of glucose decreased linearly in a time-dependent manner and was almost complete within 1 h. This experiment refutes the misconception that 6 h fast is needed to ensure gastric clearance of food consumed during the dark phase. In summary, the 6 h fasting model that is widely and routinely used in metabolic research for MTT is suboptimal due to major weight loss (>5%) and liver glycogen depletion. We therefore recommend using a 2 h fast before ITT and short fasting times <4 h before other types of MTT.

### Route of administration

The influence of the glucose administration route on glucose tolerance has been tested in C57BL/6J mice by delivering a 2 g/kg glucose bolus either by intraperitoneal injection (i.p. GTT) or orally (oGTT) via gavage after 6 h fasting^2^. i.p. GTT induced a massive unphysiological increase in blood glucose levels, nearly reaching 35–40 mM (~600–700 mg/dl) even under standard chow diet. Interestingly, glucose tolerance following i.p. GTT seemed to be only moderately altered in HFD-fed mice compared with chow diet-fed mice. The insulin response was similar between chow diet and HFD. By contrast, the oGTT produced a more relevant pathophysiological increase in blood glucose levels, reaching 25 mM in HFD-fed mice at 15 min. Glucose and insulin excursions during the oGTT were markedly different between HFD-fed and chow-diet mice. Thus, oGTT produced a greater insulin response compared with i.p. GTT at 15 min post-glucose load. Small et al. made similar observations, demonstrating that oral administration of glucose (oGTT) induced a more robust blood insulin secretion response in mice compared with i.p. administration, which was driven by C-peptide secretion and incretin response, particularly through an increase in gastric inhibitory polypeptide levels^34^. By contrast, plasma insulin, C-peptide, gastric inhibitory polypeptide and glucagon-like peptide 1 remain virtually unchanged in response to i.p. glucose injection. Endogenous glucose production (EGP) was not suppressed in mice after i.p. GTT, presumably due to the lack of insulin response. In addition, rates of exogenous glucose appearance ([2-^2^H] and [6,6-^2^H_2_] glucose isotopes) into circulation were reduced in HFD-fed mice compared with lean mice after i.p. GTT, which was not observed after oral gavage^34^. Differences in the rate of exogenous glucose appearance between groups complicate the interpretation of i.p. GTT, and these differences are probably due to direct injection into the visceral fat, reduced diffusion or reduced local absorption by capillaries in the peritoneal cavity. Bypassing the oropharyngeal sphere generally removes the influence of nervous signals (vagal afferents) and gastrointestinal hormones. Thus, we recommend using the oral route of glucose administration to avoid possible artifacts originating from a nonphysiological route^34^.

### Bolus dose

The dose of glucose is a major variable that is not sufficiently taken into consideration in the GTT design. Andrikopoulos et al. demonstrated more than 15 years ago that the glucose dose must be sufficiently high to provoke frank glucose excursions during an oGTT^2^. Using an oGTT of 2 g/kg body weight after a 6 h fast, they found marked differences in glucose and insulin responses between lean and obese mice, while blood glucose and insulin responses were virtually unchanged between lean and obese mice at lower glucose bolus doses. With the assumption that LBM is the main site of glucose disposal in mice as in humans, McGuinness et al. recommended in 2009 to normalize the glucose dose on the basis of LBM rather than total body weight^35^. We reassessed this issue using our previously published data from insulin-stimulated glucose uptake experiments performed in lean and obese mice in vivo^36–38^, in which objective measures of LBM and FM were obtained by EchoMRI (Table 2). By assuming that muscle represents approximately 50% of LBM, we estimated that glucose disposal was predominantly accounted for by skeletal muscles in lean mice (83%). However, given that insulin also induces nonnegligible rates of glucose uptake in FM^39^, and that FM can increase up to 4–5 fold in obese mice, we estimated that glucose uptake in FM reached ~44% of total glucose disposal in obese mice (Table 2). Thus, contrary to humans, FM is highly metabolically active in mice and a substantial contributor of total glucose disposal. Therefore, we strongly recommend that the glucose dose be normalized to total body weight rather than LBM, particularly in obese mice. Normalization of the glucose dose to LBM will inevitably lead to inappropriate glucose excursions and to erroneous conclusions particularly if strong differences in FM exist between animals.

**Table 2:** The relative contribution of muscle and adipose tissue glucose uptake in lean and obese mice

Body composition	BW	LBM	FM	Muscle
**Lean mouse**				
Mass (g)	32	23	4	~11.5
Absolute GU (µmol/min)	–	–	0.85	4.2
Relative GU (%)	–	–	~17%	~83%
**Obese mouse**				
Mass (g)	49	25	18	~12.5
Absolute GU (µmol/min)	–	–	3.8	5.0
Relative GU (%)	–	–	~44%	~56%

GU, glucose uptake. Body composition was objectively measured in one lean and one obese mouse. We estimated muscle mass as 50% of LBM. Absolute rates of GU were derived from personal previously published data^36–38^. In this model, the calculations were based on the assumption that total glucose disposal was accounted for by skeletal muscles and adipose tissue only.

In 2021, Bruce et al. investigated the divergent metabolic response to oGTT between mice and humans using [6,6-^2^H_2_] glucose, a stable isotope of glucose^40^. While humans experienced a sustained insulin response and robust suppression of EGP, C57BL/6J mice presented only transient changes in insulin response and poor suppression of EGP. This result might be due to the handling stress of the procedure. Thus, mice seem to be highly reliant on glucose effectiveness, for example, the ability of hyperglycemia to regulate glucose uptake and production under basal/constant insulin concentrations, to clear exogenous glucose compared with humans during an oGTT^40^. This high level of glucose effectiveness in mice probably results from the inherently high metabolic rate that small mammals require to preserve body temperature. This study highlights that handling stress and mouse housing temperature are others important confounding variables in metabolic research.

### Handling stress

Several steps of the MTT protocols requiring human intervention (such as transition to fasting, cage change and baseline blood glucose measures) might be particularly stressful to mice. Kennard et al. reported initial increases in blood glucose concentrations within the first 1–2 h of the fast in all their protocols, even when the mice were not handled, with the only human intervention involving removing the food from the lid of the cage^19^. Contrary to humans, blood withdrawal in rodents necessitates restraint, which inevitably inflicts a high level of stress. Ghosal et al. studied the influence of the classical ‘tail-picked’ versus the ‘cup’ handling method on glucose tolerance in CD1.C57BL/6J mice naive to any specific handling. They showed that cup handling reduced anxiety-like behaviors as well as blood glucose excursions during i.p. GTT^41^. Therefore, mouse cup handling might help reduce background stress and improve data quality when performing MTT in mice. In 2023, Pedersen et al. investigated the influence of handling stress on acute glucose regulation in conscious Sprague-Dawley rats given an oGTT of 2 g/kg (ref. ^42^). Blood was sampled frequently up to 90 min after challenge by handheld sampling (HS) or by automated sampling (AS). In the HS experiment, blood was withdrawn by restraint and sublingual vein puncture; 2 weeks later, samples were obtained by AS through an implanted catheter in a carotid artery, allowing sampling without disturbing the animals. In average, glucose excursion during oGTT was 17% higher in the HS compared with the AS experiment. This difference was probably explained by greater plasma levels of adrenocorticotropin (×2.4), corticosterone (×3.6) and glucagon (×3.2) during the oGTT in the HS condition. Although sampling from an arterial catheter has the advantage of providing vascular access during an experiment without introducing handling stress, catheterization of the carotid artery is invasive and can result in the loss of animals before the study. Therefore, it is not possible to use arterial sampling as a standard method for blood acquisition for routine MTT. In summary, protocol refinements should aim to minimize handling stress as much as possible to increase clinical translatability of test results.

### Cage change and bedding

Rodent husbandry requires careful consideration of environmental factors that may impact colony performance and subsequent physiological studies. A 2022 study demonstrated that whole-cage change remarkably impairs glucose tolerance in male and female C57BL6/J mice compared with bedding retention cage change (BRCC) or no cage change (NCC)^19^. BRCC consisted of mice being placed in a new cage with new wood chippings, but the bedding and enrichment from the old cage was retained and placed in the new cage. NCC consisted of mice not being handled, with food being removed from the cage lid only. Interestingly, BRCC tended to also decrease glucose tolerance in male mice compared with NCC^19^. In addition, Sveeggen et al. reported that corncob bedding containing digestible hemicelluloses, trace sugars and fibers—in part due to its ingestion by mice—could impact vascular measurements and fasting blood glucose levels compared with pulp cellulose bedding^43^. This is in agreement with another study in C57BL/6NCrl mice showing higher blood glucose levels after a 14 h overnight fast with corncob compared with hardwood bedding with or without enrichment^44^. Thus, we suggest that bedding information be routinely included in published methods to improve reproducibility.

### Housing temperature

Housing temperature has emerged as a main confounding variable in metabolic research^45^. Although various studies have defined that thermoneutrality is reached between the ambient temperature of 28 °C and 32 °C for laboratory mice, most of the animal care facilities have ambient temperature set at 21–23 °C (ref. ^46^). This discrepancy produces a major thermal stress associated with a 2-fold increase in daily energy expenditure^47^, which also has a major effect on blood glucose and insulin levels^48^. While some studies have demonstrated that mice housed at thermoneutrality have an increased susceptibility to diet-induced obesity and type 2 diabetes compared with mice housed at standard room temperature^49^, others have failed to observe a noticeable effect of thermoneutrality on western diet-induced steatohepatitis in mice over 16 weeks^29^. Yet, the investigators reported lower blood glucose excursions during i.p. GTT at thermoneutral temperature in both male and female C57BL/6J mice under standard chow and western diet compared with room temperature^29^. This finding is consistent with our own observations that housing at thermoneutrality reduces glucose excursions during oGTT in mice^45^. In addition, housing temperature may have a substantial influence on glucose tolerance and sensitivity to fasting before the MTT. We and others have demonstrated that mice, contrary to humans, experience rapid time-dependent weight loss in response to fasting. Even short-term fasts are associated with major weight loss at ambient temperature, a phenomenon that is attenuated by housing mice at thermoneutrality^31,48^. Therefore, mice are very sensitive to food deprivation under thermal stress owing to the effort to maintain euthermia. Overall, housing mice at thermoneutrality could help maximize the scientific outcomes of experimental studies and the translation to human physiology. For better translatability of test results, it would be ideal to perform MTT both at ambient temperature and at thermoneutrality whenever possible.

### Light–dark cycles and time of day

Most investigators perform MTT work during daytime (light phase), typically switching mice to fasting at 7:00, before the test. Interestingly, we observed that body weight and blood glucose levels measured in nonfasted mice housed at 21 °C did not change throughout the day over a 24 h period^31^. This finding suggests that mice eat during the daytime to maintain a stable body weight and defend their body core temperature. Our data are consistent with another study demonstrating that restricted feeding during the light phase in a long day photoperiod had no effect on food intake and body weight^50^. In this study, the authors also showed that mice fed during the light phase while on long day length had improved glucose tolerance and whole-body insulin tolerance when tested 2 h after lights on compared with mice kept on the short photoperiod. A clear rhythm of glucose tolerance was observed in mice fed during the light period with maximal glucose tolerance just before the expected presentation of food and minimal tolerance 2 h before lights off. By contrast, no rhythm in glucose tolerance was observed in the dark-fed mice, but maximal glucose tolerance occurred 2 h before lights off. These findings suggest that circadian rhythms facilitate metabolic control and can influence glucose tolerance. Since lights turn on at 7:00 in standard animal care facilities, performing MTT during daytime shortly after lights turn on would be adequate. For reproducibility and cross-comparison between experiments, MTT should be performed at the same time of day in the same experimental setting.

### Blood sampling

Anesthesia can be used to avoid stress of gavage and blood sampling when performing MTT. Windelov et al. studied the influence of anesthesia on blood glucose and insulin dynamics in female C57BL/6J mice during oGTT 2 g/kg after an overnight fast (16–18 h)^51^. Their results are unequivocal and show that the four different anesthetic regimens tested (hypnorm/midazolam, ketamine/xylazine, pentobarbital and isoflurane) markedly impaired glucose tolerance compared with saline/no anesthesia^51^, probably via inhibition of gastrointestinal tract motility. These authors therefore conclude that anesthesia should not be used when performing metabolic studies in mice^51^.

To perform MTT, multiple blood samples need to be taken typically within a 2 h period from conscious mice. The method for blood sampling can also affect blood glucose response. Christensen et al. examined the effects of four distinct methods for blood sampling in conscious C57BL/6J mice during an oGTT. Among the four tested methods, blood sampling after amputation of the tail tip had minimal effects on blood glucose levels (<1 mM) compared with periorbital puncture or lateral tail incision^52^.

### Litter size

Litter size is a biological variable that strongly influences adult physiology in rodents. For instance, several studies used models of litter size reduction during lactation (two to four pups per female lactating dam) to experimentally induce accelerated neonatal growth and early overweight/obesity in rodents^53–55^. Rodents suckled in small litters often develop adult obesity and obesity-related metabolic diseases, including insulin resistance, glucose intolerance, cardiovascular disease and altered sexual maturation. By contrast, when rodents are experimentally assigned to large litters during the suckling period (ten or more pups per female lactating dam), food intake, neonatal growth and adiposity are reduced, while insulin sensitivity is improved^56,57^. In 2023, Parra-Vargas et al. discussed this issue, showing that litter size in rodents is also inversely correlated with various metabolic parameters such as body weight, FM, blood glucose and insulin levels^58^. Thus, the authors recommended to explicitly report this important biological variable in research articles for ensuring research transparency, reproducibility and interpretation^58^.

### Data analysis

One confounding issue to consider with MTT is that the prevailing glucose level will influence the rate and magnitude of change in glucose levels (for instance, increase during oGTT and drop during ITT). In addition, as glucose concentration falls during ITT, it can activate insulin counter-regulation. The glucose threshold for increased counter-regulatory hormone release in mice is ~80 mg/dl (ref. ^25^). Thus, high bolus doses of insulin (>1 U/kg) should be avoided during ITT as they will inevitably induce severe hypoglycemia and promote a counter-regulatory response through stress hormones (epinephrine, norepinephrine, glucagon and corticosterone)^25^. Insulin half-life is quite fast in mice (~10 min)^59^. Thus, the majority of insulin will normally be cleared 30 min after injection. Therefore, differences in glucose concentration observed after the initial fall during an ITT may not relate to insulin action. To circumvent these issues, researchers should interpret glucose kinetics using an incremental area under the curve (iAUC) during GTT or the area above the curve (AAC) during ITT, also called by others inverse AUC (Table 3). These measures allow to isolate the true influence of glucose dynamics independently of the baseline value^1^. Another important variable in experimental research is sample size. Although sample size calculations are not always routinely performed in experimental research, this practice could help reach more robust and statistically powered results and reduce the number of mice necessary to test a scientific hypothesis.

**Table 3:** Proposed recommendations to perform MTT in laboratory mice

MTT	Fasting time	Route	Dose amount	Timing (min)	Data analysis	Observations
GTT	<4 h	Oral	1.5–2.0 g/kg BW	90–120	iAUC	i.p. possible to bypass the GI tract
ITT	2 h	i.p.	0.50–0.75 U/kg BW	90–120	AAC	0.5 U/kg for chow diet; 0.75 U/kg for HFD
PTT	<4 h	i.p.	2 g/kg BW	90–120	iAUC	–
GlyTT	<4 h	i.p.	2 g/kg BW	90-120	iAUC	–
ITT 2DG*	2 h	i.p.	3 U/kg BW; 0.4 µCi/g BW	15	Tissue 2DG*	–
GTT 2DG*	<4 h	Oral	1.5–2.0 g/kg BW; 0.4 µCi/g BW	45	Tissue 2DG*	i.p. possible to bypass the GI tract
Insulin signaling	2 h	i.p.	3 U/kg BW	10	P-protein	–

Researchers should carefully choose the mouse strain depending on their scientific question, although the widely used C57BL/6J mouse strain seems to be appropriate to study glucose metabolism. Researchers should work on conscious mice, during the morning light phase, ideally with mice housed at temperatures close to thermoneutrality (>25 °C). Mouse handling should be limited before the MTT, by simply removing food from the lid of the cage (NCC) and performing blood sampling at the tip of the tail, preferably using cup mouse handling.

GI, gastrointestinal; GlyTT, glycerol tolerance test; P-protein, phospho-protein level; PTT, pyruvate tolerance test.

## More complex metabolic tests

### Tissue-specific glucose uptake

To determine 2-deoxyglucose uptake in various tissues, we previously performed a bolus injection of 2-[1,2-^3^*H*(*N*)]deoxy-d-glucose (2DG*; 0.4 μCi/g body weight) and insulin (3 mU/g body weight) intraperitoneally in 2 h-fasted mice (ITT 2DG*; Table 1)^37,38^. Mice were killed 30 min post-injection, tissues were surgically removed, snap frozen and stored at −80 °C until further processing. Tissue-specific accumulation of 2DG-6-phosphate was determined as previously described, with minor modifications^39^. The total of the [^3^H]-radioactivity found in 2DG-6-phosphate was divided by the mean specific activity of glucose at 30 min to obtain the tissue-specific metabolic clearance index (Rg; μmol per 100 g of wet tissue per minute). Depending on the scientific question, it is possible to administer a mixed bolus of 1.5–2.0 g/kg of glucose and 2DG*. Others have used a similar protocol with a lower bolus dose of insulin (0.5 U/kg body weight)^60^. More recently, Cutler et al. used a slightly modified version of ITT 2DG* with dual injection of 2-[^14^C]DG 40 min before a mixed bolus of 0.75 U/kg LBM of insulin combined to 2-[^3^H]DG^30^. This new technique offers the unique advantage of measuring both basal and insulin-stimulated glucose uptake in a given tissue for each individual animal. This assay may help to reduce the number of mice required to demonstrate statistical significance to detect insulin resistance, relative to other established approaches using a single tracer. The dual tracer test may be a valuable addition to the metabolic phenotyping toolbox.

### Tissue-specific insulin signaling

Previous studies have used 10 U/kg of insulin to assess tissue-specific insulin signaling in various tissues in mice after a 6 h fasting (Table 1). Tissues were collected 10 min post-injection to determine phospho-protein levels of insulin receptor, insulin receptor substrate-1 and Akt. This dose was sufficient to induce a ~7-fold increase in Akt activity in the liver and white adipose tissue, and a nearly 10-fold increase in skeletal muscle compared with mice that received saline^61^. However, it is unclear whether maximal phosphorylation levels were reached for most insulin signal molecular transducers in various tissues. In previous studies^37,38,62^, we performed a dose–response investigation of insulin bolus injection to determine the optimal insulin dose to assess tissue-specific insulin signaling. Half maximal effective concentration (EC_50_) of insulin-stimulated Akt phosphorylation was ~3.4 U/kg for soleus, ~2 U/kg for extensor digitorum longus, ~0.72 U/kg for liver and ~7.2 U/kg for brown adipose tissue. The mean EC_50_ of insulin-stimulated Akt phosphorylation in the main insulin-sensitive tissues was ~3 U/kg. Thus, when investigating tissue insulin signaling, we recommend using a bolus insulin dose of 3 U/kg (Table 3).

### Hyperinsulinemic–euglycemic clamps

The hyperinsulinemic–euglycemic clamp is a gold-standard technique to assess insulin sensitivity and glucose metabolism in vivo in conscious mice^63,64^. Berglund et al. have reported that C57BL/6J mice display an intermediate physiological response to hyperinsulinemic–euglycemic clamp compared with other strains^5^, thus suggesting that C57BL/6J mice are a suitable model for studies of glucose homeostasis. However, like any experimental technique, this method has several limitations, especially when applied to mice. Notably, the procedure is technically demanding and requires considerable expertise to perform accurately. Challenging steps include the surgical implantation of catheters and the precise administration of insulin and glucose, which precludes the widespread and routine use of this technique in less experienced laboratories. There are others experimental limitations to consider. During the clamp, the small size and limited blood volume of mice make frequent blood sampling challenging and can lead to important physiological changes if not managed carefully^26^. The frequency of blood sampling required to accurately measure glucose and insulin levels can also be stressful for animals. For instance, a study by Ayala et al. showed that sampling 100 µl of blood from the tail tip of mice resulted in elevated catecholamines and increased basal glucose levels compared with arterial sampling. By contrast, catecholamines levels were not elevated when taking small samples (~5 µl) from the tail tip^26^. Finally, achieving and maintaining a true steady-state condition (constant blood glucose levels) during the clamp can be difficult, and any fluctuations can affect the accuracy of insulin sensitivity measurements.

## Limitations

Unfortunately, none of the technical reports examined and discussed here used similar procedures for performing oGTT. In most studies, fasting duration varied between 6 and 18 h. While the oral route of administration of glucose was predominant among studies, the bolus dose was ranging between 1 and 2 g/kg and normalized either to BW or to LBM. Ideally, all potential confounding factors previously enumerated should be tested within one experiment on the same batch of mice using a standardized procedure for oGTT.

## Conclusions

We here comprehensively reviewed and critically examined previous guidelines and recent technical reports to discuss various methodological aspects to consider to optimally perform MTT in laboratory rodent models. We further provide revisited guidelines on what we believe are the best practices to perform these metabolic tests in laboratory mice (Table 3). We thus urge authors, reviewers and editors to give up erroneous practices such as overnight fasting before MTT in mice. We also provide practical information for protocol refinements and possible reduction of the number of laboratory mice used in metabolic research. We hope this work will help scientists reach more consensual guidelines for maximizing the relevance and clinical translation of mouse models in metabolic research. We also think this Review will be helpful to increase animal welfare and advance the 3Rs (reduce, replace, refine) in metabolic research.
